# Association of polymorphisms in bone morphogenetic protein receptor-1B gene exon-9 with litter size in Dorset, Mongolian, and Small Tail Han ewes

**DOI:** 10.5713/ajas.18.0541

**Published:** 2019-02-09

**Authors:** Jianlei Jia, Qian Chen, Linsheng Gui, Jipeng Jin, Yongyuan Li, Qiaohong Ru, Shengzhen Hou

**Affiliations:** 1 Key of laboratory of Plateau Ecology and Agriculture, Qinghai University, Xining, Qinghai 810016, China; 2 Department of Animal Science, College of agriculture and Animal Husbandry, Qinghai University, Xining, Qinghai 810016, China; 3 Department of Animal Science, College of Animal Science and Technology, Gansu Agricultural University, Lanzhou Gansu, 730070, China; 4 Animal Husbandry and Veterinary Station, Haidong, Qinghai 810700, China

**Keywords:** Sheep, Bone Morphogenetic Protein Receptor-1B (*BMPR-1B*) Gene, Polymorphisms, Polymerase Chain Reaction–Single-Strand Conformation Polymorphism (PCR-SSCP), Litter Size

## Abstract

**Objective:**

The present study was to investigate the association of polymorphisms in exon-9 of the bone morphogenetic protein receptor-1B (*BMPR-1B*) gene (C864T) with litter size in 240 Dorset, 232 Mongolian, and 124 Small Tail Han ewes.

**Methods:**

Blood samples were collected from 596 ewes and genomic DNA was extracted using the phenol: chloroform extraction method. The 304-bp amplified polymerase chain reaction product was analyzed for polymorphism by single-strand conformation polymorphism method. The genotypic frequency and allele frequency of *BMPR-1B* gene exon-9 were computed after sequence alignment. The χ^2^ independence test was used to analyze the association of genotypic frequency and litter size traits with in each ewe breed, where the phenotype was directly treated as category.

**Results:**

The results indicated two different banding patterns AA and AB for this fragment, with the most frequent genotype and allele of AA and A. Calculated Chi-square test for *BMPR-1B* gene exon-9 was found to be more than that of p value at the 5% level of significance, indicating that the population under study was in Hardy-Weinberg equilibrium for all ewes. The χ^2^ independence test analyses indicated litter size differences between genotypes was not the same for each breed. The 304-bp nucleotide sequence was subjected to BLAST analysis, and the C864T mutation significantly affected litter size in singletons, twins and multiples. The heterozygosity in exon-9 of *BMPR-1B* gene could increase litter size for all the studied ewes.

**Conclusion:**

Consequently, it appears that the polymorphism *BMPR-1B* gene exon-9 detected in this study may have potential use in marker assisted selection for litter size in Dorset, Mongolian, and Small Tail Han ewes.

## INTRODUCTION

Sheep farming is of major economic importance and China leads the world in both sheep numbers and breeds. The sheep industry has become the primary source of income for small and marginal farmers because it requires minimum resources. Qinghai province is one of the five pastoral areas in China. Sheep farming is the important pillar industry. With the development of ecological animal husbandry, reducing the sheep number while increasing economic benefits are the current development trend of Qinghai province sheep industry. The most direct way is to increase the ewes’ reproductive rate. However, sheep production suffers from a major constrain as the majority of sheep breeds, Dorset and Mongolian, have low litter size excepting Small Tail Han sheep and Hu sheep in Qinghai province (China).

Reproductive traits are quantitative in nature and controlled by multiple genes, loci and interactions [1]. Some mutations in bone morphogenetic protein receptor-1B (*BMPR-1B*), bone morphogenetic protein-15 (*BMP15*), and growth differentiation factor-9 (*GDF9*) genes are reported to increase ovulation rate [2]. Some studies have indicated that the ovulation rate and litter size can be genetically regulated by a set of different genes, called as fecundity (*Fec*) genes [3]. *BMPR-1B*, *BMP15*, and *GDF9* genes belong to the large family of transforming growth factor-β (*TGF-β*), and are located on chromosomes 6, X and 5 in sheep, respectively [2]. Also, several studies have been carried on goats [4,5].

The *BMPR-1B*, one of the oocyte-derived members of transforming growth factor-β family has played an imperative role in follicular growth and ovulation [6]. The *BMPR-1B* gene was the first major gene associated with reproductively in sheep [7,8]. There are 22 single nucleotide polymorphisms (SNPs) reported for *BMPR-1B* (http://www.ncbi.nlm.nih.gov/gene/443454), but only 4 SNPs change the amino acid sequence in the gene (G192A, A746G, G922T, and T1043C) [9,10]. A major research on the non-synonymous SNP of A746G found that the damage to the BMP system during follicle development led to increase average ovulation in Booroola Merino sheep, Cambridge sheep and Small Tail Han sheep [11–13]. Many aspects of the *FecB* gene, including reproductive endocrinology, ovary development, litter size, organ development and body mass have been studied [14,15]. At the same time, this gene has an additive effect on litter size and ovulation rate, but has negative effects on fetal growth and development and body mass during gestation. However, there are few reports on the effects of the other SNPs, especially the 18 synonymous SNPs.

The tendency for sheep producing twins or triplets lambs are the same, although there are differences in the level of gene regulation [16]. Research has shown that the non-synonymous mutation of *BMPR-1B* has a significant positive effect on reproduction performance in Dorset, Mongolian, Small Tail Han sheep, but genetic mechanism caused by mutations in *BMPR-1B* genes, which have a relationship with the average number of ovulation in sheep, is still not widely known. Keeping in view of this aspect, the present study was envisaged to investigate: i) to detect SNPs of the *BMPR-1B* gene exon-9 using polymerase chain reaction–single-strand conformation polymorphism (PCR-SSCP) and DNA sequencing methods, and ii) investigating the association of *BMPR-1B* genes mutations with litter size in ewes with single, twin, and multiple traits.

## MATERIALS AND METHODS

The study protocol was approved by the Regulations for the Administration of Affairs Concerning Experimental Animals (Ministry of Science and Technology, China, revised in 2004) and approved by the Institutional Animal Care and Use Committee (State key of laboratory of Plateau Ecology and Agriculture, Qinghai University, China, 2015).

### Animals collection and genomic DNA extraction

This study was conducted on 596 ewes (120 Dorset ewes with singles, 120 Dorset ewes with twins, 114 Mongolian ewes with singles, 118 Mongolia ewes with twins and 124 Small Tail Han ewes with multiple lambs maintained under the same feeding system at Huajia Farm (Dingxi, Gansu, China). All the ewes were 3–4 years old and they are bought to and fed at the Huajia Farm (Dingxi, Gansu, China) from 3 months of age. The ewes have a clear litter records, the same birth order (3rd birth orders), raising conditions (NRC2007) and body condition (the same breeding ewes’ weight was not significantly different).

Jugular blood samples of 596 individuals (5 mL each) were collected in vacutainer tubes containing heparin sodium as anticoagulant. Cold chain was maintained throughout the sampling process. Genomic DNA was isolated from blood cells using standard phenol-chloroform-Isoamyl alcohol method as per the standard protocol described by Ding [17]. The extracted DNA samples were assessed for quantity and purity using Nanodrop ND-1000 spectrophotometer (Thermo Fisher Scientific, Wilmington, DE, USA), while visual confirmation of the DNA integrity was also assessed by running on 1% agarose gel.

### Polymerase chain reaction amplification

Genomic DNA samples of 596 ewes were adjusted to a concentration of 50 ng/μL and exactly 2.5 μL of each DNA sample was used as template for PCR. Amplification procedure for *BMPR-1B* gene exon-9 of sheep has been standardized which yielded consistent and specific amplification. Genomic DNA was amplified using primer sequences (F: 5′-TCTTGGGCTT CATTGCTGCCGAT-3′ and R: 5′-TAAACTTAACAGCCAA GCCCAGGTC-3′) as described by elsewhere (GenBank accession number: NM_001009431.1).

The amplification reaction conditions were carried out using 30 cycles at 94°C for 3 min, followed by 94°C for 30 s, 56°C for 30 s, 72°C for 30 s, followed by 72°C for 10 min. The amplified products were consistent with the target fragments and had a good specificity on 2% agarose gel electrophoresis, which could be directly analyzed by SSCP.

Finally, according to the distribution of *BMPR-1B* gene exon 9 of Dorset sheep, Mongolia sheep, and Small Tail Han sheep in sheep, the amplified fragments were identified.

### Single-strand conformation polymorphism analysis

The study of sequence variation in exon-9 region of *BMPR-1B* gene was achieved by SSCP as was described by Wang et al [18] with minor modifications. About 3 μL PCR product and 10 μL SSCP loading buffer dye (Bio-Rad, Hercules, CA, USA) were taken in a 0.5 mL tube. Each sample was denatured at 98°C for 10 min. After denaturation it was kept immediately in ice for 10 min to maintain it as single strand DNA for its conformation. The denatured PCR products were run in a non-denaturing 12% polyacrylamide gel for 8 to 10 h at 8 V/cm in room temperature. After electrophoresis, SSCP gels are fixed and stained in a solution containing 10% ethanol, 0.5% acetic acid, and 0.2% silver nitrate as the method described by Li et al [19] with minor modifications to identify the DNA sequence variations. The silver stained gel was kept on trans-illuminator and SSCP variants were recorded for genotyping of ewes.

### FecB genetic detection

We detected *BMPR-1B* gene of 746 loci by Forced PCR-restriction fragment length polymorphism (FRLP) technology. We used enzyme cleavage by Ava II restriction endonuclease, and detected by 3% agarose gel electrophoresis.

### DNA sequence analysis

The PCR products were purified using TIANGEN PCR purification kit (Beijing, China) to remove primer dimers and other PCR ingredients before custom sequencing following manufacturer’s instructions. PCR products showing different banding patterns on SSCP gel were selected for sequencing. Custom sequencing of the purified PCR products from four selected animals was performed in both directions using DNA sequencing service provided by Shanghai Invitrogen Biotechnology Ltd. Co. (Shanghai, China). Sequence alignments, translations and comparisons were carried out on the BLAST website (http://blast.ncbi.nlm.nih.gov/Blast.cgi) and Chromas 2 and MEGA 5.05 software.

### Statistical analysis

The genotypic frequency, as well as the frequency of different alleles of *BMPR-1B* gene exon-9, was computed after sequence alignment. Chi-square test was used for statistical analysis in GraphPad Software (GraphPad Software, Inc., La Jolla, CA, USA).

The χ^2^ independence test was used to analyze the association of genotypic frequency and litter size traits with in each ewe breed, where the phenotype was directly treated as category. Tests of hypotheses were done using t-tests at p<0.05 with p<0.10 considered a trend.

## RESULT

### Polymerase chain reaction amplification

**Figure 1:**
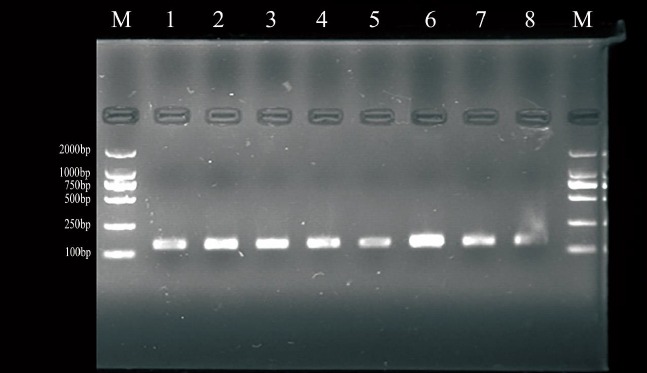
PCR amplification of FecB. PCR, polymerase chain reaction; FecB, fecundity Booroola. M was marker (DL2000), 1 to 8 were PCR amplification fragments of FecB.

**Figure 2:**
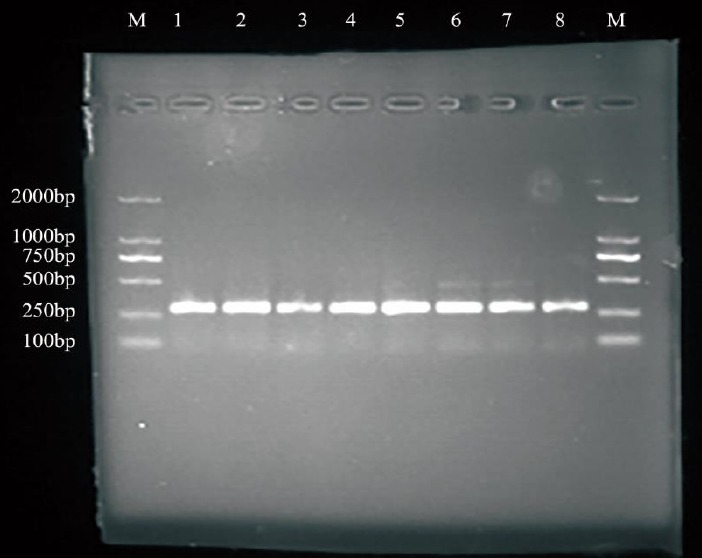
PCR amplification of *BMPR-1B* gene exon-9. PCR, polymerase chain reaction; *BMPR-1B*, bone morphogenetic protein receptor-1B. M was marker (DL2000), 1 to 8 were *BMPR-1B* gene Exon-9 PCR amplification fragments.

The amplifications resulted in a product size of 140-bp (*FecB* gene) and 304-bp (*BMPR-1B* gene exon-9) length on 2.5% agarose gel electrophoresis when visualized under the UV transilluminator in all ewes (Figures 1, 2). This product was directly used for SSCP analysis.

### FecB genetic detection

**Figure 3:**
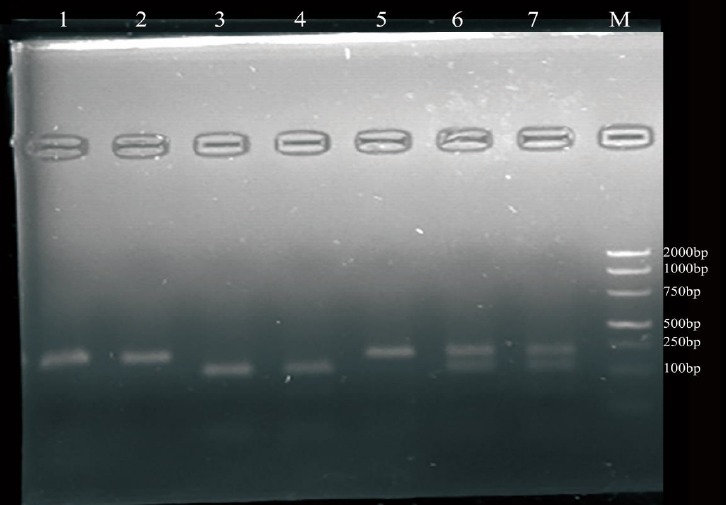
Polymerase chain reaction-restriction fragment length polymorphism of *FecB* gene. *FecB*, fecundity Booroola. 1, 2, 5 were genotypes ++; 3, 4 were genotypes BB; 6, 7 were genotypes B+; M was DNA DL2000 Ladder marker.

We selected ++ genotypes of *FecB* gene by PCR-FRLP technology (Figure 3) to identify genetic variants of *BMPR-1B* gene exon-9 in a total of 596 ewes.

### Polymerase chain reaction–single-strand conformation polymorphism analysis

**Figure 4:**
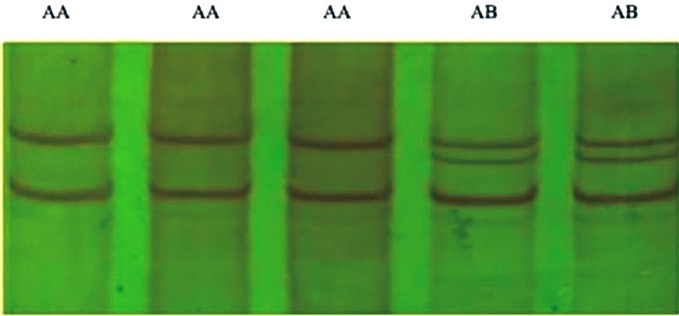
Polymerase chain reaction-single-strand conformation polymorphism of bone morphogenetic protein receptor-1B gene exon-9. AA was wild-type, AB was polymorphic-type.

PCR-SSCP technique was used to identify genetic variants of *BMPR-1B* gene exon-9 in a total of 596 ewes. The results revealed two SSCP variants for PCR products in non-denaturing 12% polyacrylamide gel, which were arbitrarily assigned as AA and AB type of variants (Figure 4). The AA pattern showed two bands whereas AB pattern showed three bands.

It was found that out of all studied ewes with ++ genotypes of *FecB* gene, among 120 singletons Dorset ewes, 98 samples had AA genotype and 22 samples had AB genotype, among 120 twins Dorset ewes, 78 had AA genotypes and 42 had AB genotypes, in 114 singletons Mongolia ewes, 94 had AA genotypes and only 20 had AB genotypes, in 118 twins Mongolia ewes, 70 had AA genotypes and 48 had AB genotypes, and in 124 multiples Small Tail Han ewes, 84 had AA genotypes and 40 had AB genotypes in *BMPR-1B* gene exon-9 (Table 1). However, among the animals examined, BB genotype could not be detected.

### Sequence analysis of *BMPR-1B* gene exon-9

The PCR products corresponding to each of the different PCR-SSCP alleles/patterns were selected and sequenced using the forward and reverse primer to detect variations at the nucleotide level. The sequence obtained was subjected to BLAST (www.ncbi.nlm.nih.gov/BLAST) analysis to as certain that sequences were of *BMPR-1B* gene exon-9.

**Figure 5:**
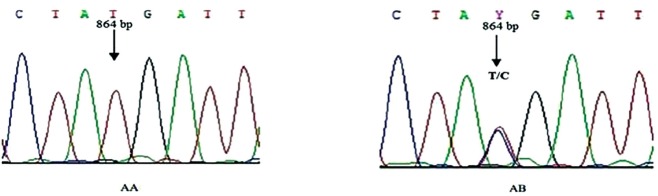
DNA sequences of AA and AB genotypes of bone morphogenetic protein receptor-1B gene exon-9.

The results obtained by DNA sequencing showed a transition of T→C at position 864 of the amplified fragment of *BMPR-1B* gene exon-9 with UCA changed to UCG (bothencode Serine) (Figure 5).

### Genotype distribution, genotype and allele frequencies

The distribution of the different genotypes for the *BMPR-1B* gene exon-9 in all ewes studied by means of PCR-SSCP is presented in Table 1.

The χ^2^ independence test was used to analyze the association of genotypic frequency and litter size traits with in each ewe breed. The results showed (Table 2) that differences between the singletons and twins ewes in AA and AB genotypic frequency were significant, respectively (χ^2^ = 7.6918, p<0.05) in Dorset ewes, χ^2^ = 13.8814, p<0.05 in Mongolia ewes). There were significant differences between three litter size and multiple litter size in Small Tail Han ewes (χ^2^ = 6.2079, p<0.05).

There were statistically significant differences for B allele frequency in ewes through the χ^2^ independence test (p<0.05). This meant that B allele frequency was promoted in Dorset twin ewes (p<0.05) and Mongolian twin ewes (p<0.05). And there were no significant differences in Small Tail Han ewes in litter sizes (p = 0.1069), but there was a trend for litter size with B allele frequency.

## DISCUSSION

BMPR-1B, a member of TGF-β super family, is a major gene for ovine ovulation rate and litter size, plays a pivotal role in follicle development and litter size. Previous studies identified the A746G mutation, a loss-of-function mutation in *BMPR-1B* gene, generates a mutant protein that promotes steroid production and ovulation rate and increases litter size in Australian Merino sheep, which was named *FecB* gene (or FecB mutation of *BMPR1B* gene) [20]. The mutation of *BMPR-1B* gene (C746T) was used as the candidate gene for twining trait by SNP and differentially expressed mRNA in Mongolian ewes and Tan ewes. The reports showed that BMPR-1B had additive effects on ovulation rate and it could increase 1.5 to 2.0 for each copy in Booroola Merino sheep [21]. Meanwhile, BMPR-1B had the effect to inhibit granular cells apoptosis, prevent follicular atresia, and promote ovulation and litter size. This may be an important physiological mechanism whereby BMPR1B affects fecundity in sheep [22]. Numerous studies have indicated that the differences at the level of gene regulation cannot make the different tendency for sheep producing twins or triplets traits. Though we know a little about the follicles regulatory mechanism of BMPR-1B in single, twins and multiple prolific ewes, these properties of BMPR-1B can provide new ideas to continue studies of ewe follicular development and litter size.

In the current study, we further demonstrated that genetic variation in ewes *BMPR-1B* gene exon-9 was also associated with litter size in ewes. To our knowledge, *BMPR-1B* gene exon-9 has a modification effect on *FecB* gene. The non-synonymous mutation of BMPR-1B exon-9 can competitively combine to miR-204 to regulate the expression level of smads protein phosphorylation, so the mutated *BMPR-1B* gene exon-9 may influence the expression of oocyte meiosis to affect the litter size of ewes by other unknown mechanism [23]. Polymorphisms of *BMPR-1B* gene exon-9 were detected by PCR-SSCP and DNA sequencing, and analyzed the association with singletons, twins and multiples traits. The amplification of exon-9 of *BMPR-1B* gene resulted in a product size of 304-bp in all ewes of Huajia farm in the present study and was in good agreement with the result reported by Wang in Texel and Tibetan sheep. Similar findings were also reported by Chu in Hu Sheep breeds reared in China [21]. A point mutation (T→C) at position 864 of *BMPR-1B* gene was detected, but it did not change the amino acids sequences. There were only two genotypes (AA and AB) without BB genotype in our research, which was the same as reported in Davis GH, however, contrary to Chu’s study. The causes for this situation could be that i) BB ewes existed in all sheep breeds, but there were no BB genotype ewes in Dorset ewes, Mongolia ewes and Small Tail Han ewes. PCR-SSCP and DNA sequencing methods were not suitable to detect the polymorphisms of *BMPR-1B* gene exon-9, and the allelic frequency of B was too low to be detected in this study as the sample sizes were too small. ii) BB ewes does not exist in all sheep breeds, which could indicate that the allele B was a recessive lethal gene and BB genotype ewes could not survive. This finding was in agreement with those of Ghaderi, who also reported rare occurrence of homozygote BB in Kordi sheep and Arabic sheep, but Roy, Moradband and Abdoli reported three distinct genotypes in Bonpala, Baluchi, and Mehraban ewes respectively.

Litter size is a low heritability trait in ewes, and is controlled by a major gene and some minor-polygenes, therefore, it was very important to identify and screen candidate genes for the standardization model of sheep husbandry [24–26]. In this study, The SNP T864C locates in the exon 9 of the *BMPR-1B* gene, which did not result in changes in amino acids, but the genotype distribution of different litter size traits ewes (singletons ewes, twins ewes, and multiples ewes) had significant differences. To our knowledge, the mutation of BMPR-1B exon-9 changed codon UCA to UCG, and UCG was a rare codon, and the rare codons could influence numerous other stages of protein metabolism, such as the rare codons appearing to influence the translation rate, which in turn affects protein folding. It could hinder protein exerting normal function, the result was the same as with Roy J [27–29]. In recent years, an extensive literature has proved that the non-synonymous mutation can also influence the phenotypic expression and function of some genes. The non-synonymous mutation of homeobox protein gene exon-2 resulted in decreased levels of mRNA expression or allele-specific differences in mRNA folding could influence splicing, processing, or translational control and regulation, which is significantly associated with increased esophageal and gastric cancer risk in Chinese [30–33]. In our study, synonymous mutation could result in activation of the native splicing donor site, which results in a premature stop codon or exon skipping, yielding a shorter mRNA, and the C864T polymorphism affect ewes’ ovarian specific expressions of follicular oocyte cells and granulosa cells under the unusual condition. At the same time, C864T is in linkage disequilibrium with other common functional non-synonymous polymorphisms such as A746G. These associations may also be the results of linkage between these SNPs and other genes on the same chromosomes that have a significant effect on these production traits.

Since the genetic code is redundant, having multiple codons for many amino acids, some mutations will not alter the protein sequence encoded by a mutated gene. The change is from one codon to another for the same amino acid. These mutations are designated silent or synonymous mutations. Examples of these are seen in a number of human genetic disorders. Furthermore, these effects may play a role in codon usage evolution. Silent mutations have also been deliberately introduced into recombinant genes to improve or alter expression. According to above causes, we hypothesize that the mutation was a “Silent” polymorphism in the *BMPR-1B* gene exon-9, and it had the presence of a rare codon, marked by the synonymous polymorphism, and it also had association with different litter size of ewes (singletons ewes, twin’s ewes, and multiples ewes).

## Figures and Tables

**Table 1:** Genotype distribution, allelic and genotypic frequencies of the *BMPR-1B* gene exon-9 in ewes

Samples	No. of ewes	Genotypic frequency1),2)		Allelic frequency3)	
	
		AA	AB	A	B
Singletons Dorset ewes	120	0.82(98)	0.18(22)	0.91	0.09
Twins Dorset ewes	120	0.65(78)	0.35(42)	0.83	0.17
Singletons Mongolia ewes	114	0.82(94)	0.18(20)	0.91	0.09
Twins Mongolia ewes	118	0.59(70)	0.41(48)	0.80	0.20
Multiples Small Tail Han ewes	124	0.71(88)	0.29(36)	0.86	0.14

*BMPR-1B*, bone morphogenetic protein receptor-1B.

1) AA was wild-type of *BMPR-1B* gene exon-9, AB was mutation-type of *BMPR-1B* gene exon-9.

2) Numbers in parentheses are numbers of individuals that belong to the respective genotypes.

3) Mutant allele for *BMPR-1B* gene (B), wild type allele for *BMPR-1B* gene (A).

**Table 2:** Genetic diversity index of the *BMPR-1B* gene exon-9 in ewes

Breeds	PIC	He	Ne	p-value
Singletons Dorset ewes	0.35	0.29	1.406	0.09
Twins Dorset ewes	0.32	0.2442	1.3213	0.16
Singletons Mongolia ewes	0.37	0.3537	1.5435	0.11
Twins Mongolia ewes	0.35	0.2938	1.4136	0.13
Multiples Small Tail Han ewes	0.22	0.1352	1.1556	0.78

*BMPR-1B*, bone morphogenetic protein receptor-1B; PIC, polymorphism information content; He, heterozygosity; Ne, effective number of alleles.
